# Post-operative hemimaxillectomy rehabilitation using prostheses supported by zygoma implants and remaining natural teeth

**DOI:** 10.6061/clinics/2016(10)04

**Published:** 2016-10

**Authors:** Xing Zhou Qu, Ming Yi Wang, Hui Shan Ong, Chen Ping Zhang

**Affiliations:** Shanghai Ninth People’s Hospital, Affiliated to Shanghai Jiao Tong University, Department of Oral Maxillofacial-Head & Neck Oncology, Shanghai, China

**Keywords:** Maxillectomy, Zygoma Implant, Rehabilitation, Finite Element Analysis

## Abstract

**OBJECTIVES:**

This study aimed to evaluate the stability of prostheses supported by zygoma implants and remaining teeth for subjects who had undergone hemi-maxillectomy.

**METHODS:**

Ten patients were included in the study. Oral rehabilitation was performed using a temporary prosthesis that was supported by remaining teeth for the first three months. Then, a zygoma implant was placed to provide support for a final prosthesis in addition to the remaining teeth. Each prosthesis was tailor-made according to biomechanical three-dimensional finite element analysis results. The patients were assessed using the prosthesis functioning scale of the Memorial Sloan-Kettering Cancer Center. In addition, retention and bite force were recorded for both the temporary prosthesis and the final prosthesis.

**RESULTS:**

The mean bite force of the prosthetic first molar was increased to 69.2 N. The mean retentive force increased to 13.5 N after zygoma implant insertion. The bite force on the prosthetic first molar was improved to 229.3 N.

**CONCLUSION:**

Bite force increased significantly with the support of a zygoma implant. The use of zygoma implants in the restoration of maxillary defects improved functional outcome and patient satisfaction.

## INTRODUCTION

Microvascular free flap reconstruction, a technique that utilizes both soft and bony tissues to achieve wound closure, continues to revolutionize functional and esthetic criteria for complicated head and neck reconstructive surgeries. Although microvascular reconstructive techniques for maxilla defects have improved, limitations caused by irradiation fields without optimal recipient vessels prolong hospitalization, incur post-operative complications, cause donor-site morbidity, lead to a risk of flap failure (including through bone resorption), prolong treatment time, cause blood loss, and create financial difficulties, ultimately resulting in patient refusal 1. The above considerations have led to the use of maxillary prostheses as an alternative. However, conventional prostheses are retained and supported by remaining teeth, which lack sufficient retentiveness for phonation and deglutination and provide an unfavorable esthetic appearance (i.e., dropping during smiling) 2 Likewise, there are difficulties in preventing the displacement of prostheses during mastication, which results in the overloading of remaining natural teeth, causing them to deteriorate over time 3. The goal of using prostheses is to both restore what is lost and, most importantly, preserve what is left. To meet this goal, we strongly support the use of zygoma implant-supported maxillary prostheses. Zygoma implants better improve prosthesis retention and stability than conventional clasp techniques. We conducted the current study to evaluate the combination of using a zygoma implant and natural teeth to support conventional prostheses for post-operative hemimaxillectomy rehabilitation.

## PATIENTS AND METHODS

The present sequential retrospective study was approved by the Ethical Board of Shanghai Ninth People's Hospital. From January 2009 to December 2013, 10 patients underwent hemimaxillectomy in which reconstruction was achieved using a zygoma implant-supported prosthesis with the criterion that the opposing natural dentition remained intact. All subjects were closely followed up. In all, there were 6 cases of subtotal maxillectomy and 4 cases of total maxillectomy. The patients' characteristics are presented in Table 1.

**Figure 1:**
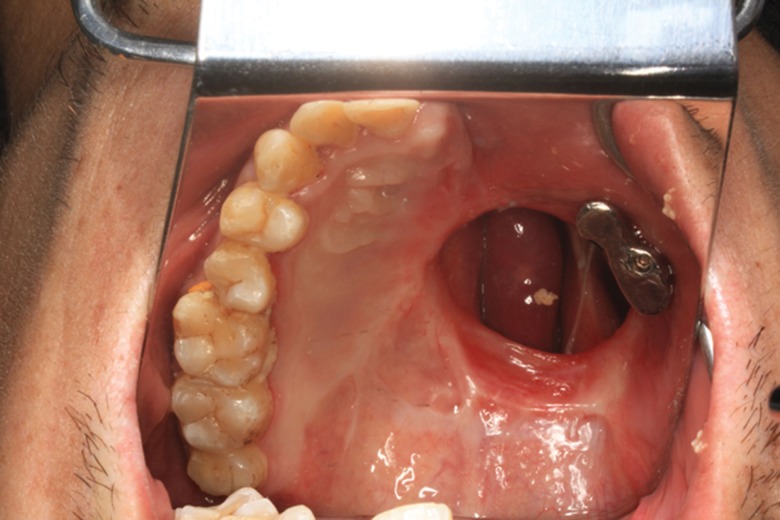
Intra-oral view of zygoma implant with magnetic abutment.

**Figure 2:**
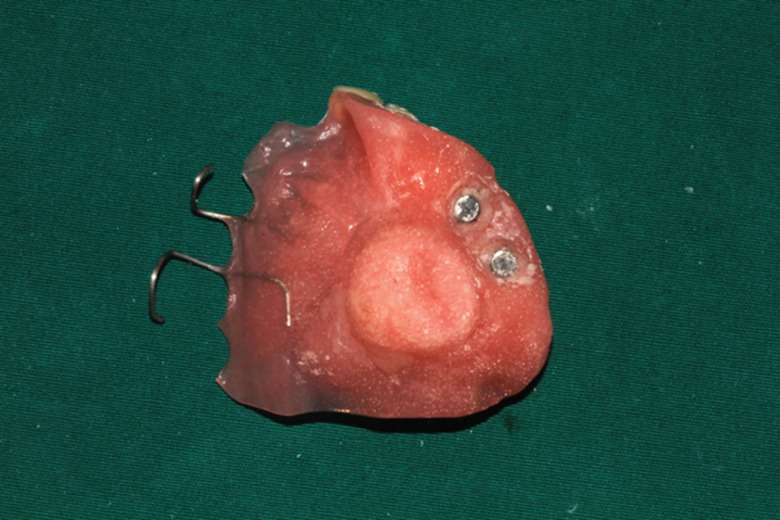
Prosthesis stability and retention substantially improved due to the foundation offered by zygoma implants and natural teeth.

The timing of zygoma implant insertion was individualized: 8 patients underwent immediate zygoma implant placement (without loading) during maxillectomy and 2 patients underwent implant placement during a second surgery. All of the prostheses (temporary and permanent) were designed by the same prosthodontist (MY Wang). Magnetic abutment was used to connect prosthesis and implant. Implant loading was postponed after confirmation of osseointegration (Figures 1 and 2) to reduce the chance of implant failure.

**Figure 3:**
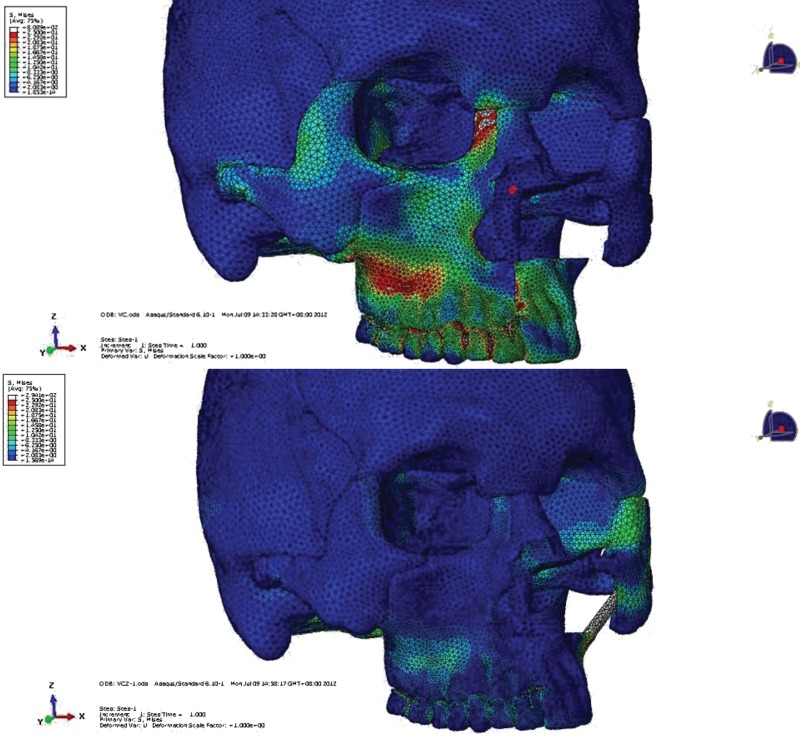
Comparison of stress distribution patterns between conventional prostheses with and without the use of a zygoma implant. For prostheses not supported by a zygoma implant, the stress distribution occurred along the remaining alveolar bone. However, for prostheses supported by a zygoma implant, masticatory stress was evenly distributed among abutment teeth, prosthesis and zygoma implant.

**Figure 4:**
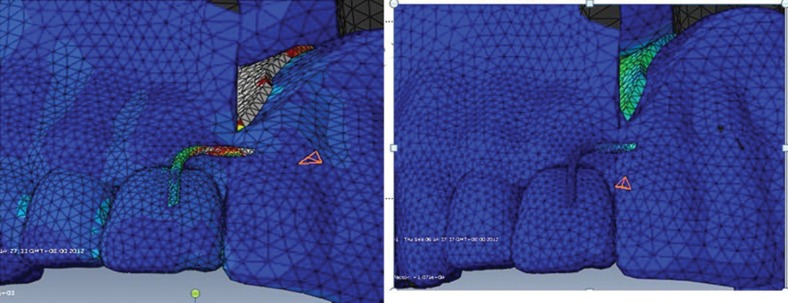
Stress on the “I” clasp was significantly decreased with the use of a zygoma implant for additional support.

Before performing the above-described procedure, CT data were gathered from a normal patient. A biomechanical three-dimensional finite element model of a unilateral maxilla was developed. This allowed stress analysis to be conducted on each prosthesis clasp. The model was widely used to analyze stress on natural teeth and prostheses in many types of jaw defects, with reproducible results 4,5. Two groups were defined: one group received prostheses with zygoma implant support, and the other received prostheses with no implant support. Stress distributions among the groups were compared (Figure 3). Prosthesis designs were than modified to achieve the most homogenous stress distribution. The use of an incisor “I” clasp was eliminated for the zygoma implant-supported prostheses (Figure 4). Retentive force was measured on a stone model using an Easy Test universal testing machine (United Kingdom). The bite force of the prosthetic first molar was measured with a T-scan III (T-scan III 6.0, Tekscan Inc, Boston, MA, USA).

The Prosthesis Functional Scale (OFS) was applied to evaluate prosthesis quality and patient satisfaction. OFS scores were recorded after the permanent prosthesis had been worn for 3 months. The OFS consists of 15 questions divided into three subcategories; the questions are answered according to the following categories: ① not at all/a little, ② somewhat, or ③ very much/extremely. All questionnaires were distributed by the same interviewer.

Two patients underwent adjuvant radiotherapy after implant placement (60 Gy, 30 times). One patient showed recurrence a year later; this patient underwent extensive resection, but the tissue surrounding the zygoma implant was not cancerous.

Statistical analysis was performed using SPSS 17.0

## RESULTS

**Figure 5:**
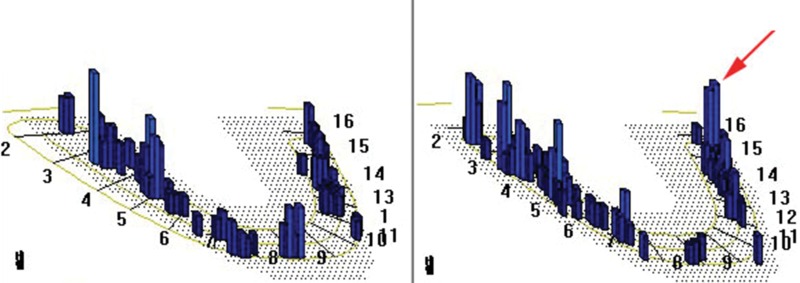
T-scan showing that the bite force over a prosthetic first molar increased if it was supported by a zygoma implant, which reflected an improvement in masticatory function.

The mean retentive force for the prostheses without implant retention was 9.5±0.12 N, and the mean bite force of the prosthetic first molar was 69.2±0.08 N. The mean retentive force increased to 13.5±0.27 N after zygoma implant insertion, and the bite force on the prosthetic first molar improved to 229.3±0.26 N. Based on the OFS results, no patients reported severe leakage. One patient complained of nasal voice without nasal regurgitation during the consumption of large quantities of water. Three patients (30%) suffered xerostomia (Grade 2 to 3) but were otherwise satisfied with the prosthesis. Statistical analysis was conducted using SPSS 17.0. The results showed that the use of a zygoma implant does not significantly improve prosthesis retention (*p=0.127*), although our patients noted subjective improvement. The bite force of the prosthetic first molar was significantly increased after implant insertion (*p=0.021*) (Figure 5). Significant improvement in routine masticatory function after implant insertion was also reported by our patients.

## DISCUSSION

The maxilla skeleton profile has both aesthetic and functional roles. Maxilla defects can therefore cause devastating functional and outcomes. The complicated three-dimensional contouring of the maxilla makes autologous tissue transfer difficult; therefore, the use of a prosthesis provides an alternative for maxillary defect rehabilitation. However, most approaches use prostheses that are retained and supported by remaining natural teeth. This strategy increases the risk for various issues, including poor retention; poor mastication; abrasive ulcerations caused by dentures; overloaded abutment teeth; leakage of saliva, liquid, and/or food; and problems with speech 6-8.

With the introduction of the zygoma implant, prosthesis retention has greatly improved, which should translate into better stability overall 9,10. Although the use of a zygoma implant to support a prosthesis is not a novel technique, as this strategy has been applied to edentulous maxilla 11, no previous studies have quantitatively proven the efficacy of this approach. We therefore conducted the current study to evaluate the efficacy of using remaining abutment teeth and a zygoma implant-supported prosthesis in patients undergoing hemi-maxillectomy.

FEA is a digital technique that is widely used in the fields of engineering and biomechanics. The process of analysis is direct and produces clear results, which allows the investigator to assess and examine all regions of interest 12. In our FEA model, we compared stress distributions between traditional prostheses supported by remaining teeth and prostheses supported by both zygoma implants and remaining teeth. We noticed that the stress on the denture base and some clasps was reduced after zygoma implant insertion. According to our stress analysis, the use of an incisor “I” clasp can be eliminated, which leads to improved aesthetics and avoids unnecessary loading on the incisor. Although the number of clasps used with the modified prostheses was less than those used with the traditional prostheses, the retention of the modified prostheses with magnet abutment was improved.

Magnet abutment technology is constantly improving and provides a useful method for attaching dental prostheses to osseointegrated implants 13,14. In our study, two patients received two zygoma implants each. In both cases, a magnet was attached to the combined armature on the dental implants placed in the zygomatic bone, which prevented problems caused by non-parallel implants. Other precision attachments, such as ball-socket attachments, are also suitable to connect an implant and prosthesis if only one zygoma implant is in place.

The prostheses in our patients were supported by remaining teeth as well as zygoma implants. Implant retention greatly improved prosthesis stability, allowing loading forces that act on implants and remaining teeth to be homogenously distributed. This reduced pressure and friction on adjacent soft tissues and improved overall patient satisfaction. Additionally, the development of traumatic ulcers on oral mucosa was prevented through the improvement of prosthesis stability. Using a T-scan III to test bite force showed that bite force increased significantly after zygoma implant insertion. Prosthesis stability is critical to the functional ability of a prosthesis, and our patients claimed to experience great improvement in mastication after implant insertion. Additionally, leakage was reduced. All patients were very satisfied with the treatment results. Although the use of zygoma implants was found to be highly advantageous, some potential risks, such as implant failure and implantitis, are associated with this methodology 15.

Implant fixation within irradiated fields is contraindicated because studies have revealed a higher failure rate in irradiated patients 16,17. Osteoradionecrosis is related to radiation dose, fractioning dose, interval between radiation and implant insertion and overlying soft tissue condition. Zygoma implantation is advantageous for regions not centered within a radiation field because bone marrow remodeling does not occur when the radiation dose does not exceed 50 Gy 18. Irradiated bones do undergo repair and neovascularization, but these changes require a minimum of 6 months, which indicates that a postoperative waiting period must be followed 18. Overall, the use of a zygoma implant-supported prosthesis is a good alternative for patients in whom the use of a microvascular free flap is contraindicated.

One limitation of the current study is the small sample size, which makes it difficult to generalize the results. Additionally, one concern associated with immediate implant placement is the potential for tumor recurrence. It should be noted that the goal behind using a zygoma implant is rehabilitation; in cases in which the zygomatic arch must be avoided due to oncological concerns, this technique is contraindicated. Therefore, case selection is very important, which should be based on close follow-ups after cancer ablation. However, for eligible patients, the use of a prosthesis shows a clear advantage compared to free flap reconstruction.

## AUTHOR CONTRIBUTIONS

Qu XZ wrote the manuscript. Wang MY and Ong HS performed the experiments and collected the data. Zhang CP designed the study and approved the submission.

## Figures and Tables

**Table 1:** Patient clinic data.

Patient	Age	Gender	Primary Lesion	Defect	Implant Number	Radiation
1	39	M	pleomorphic adenoma in palate	R subtotal maxilla	2	No
2	67	F	mucoepidermoid carcinoma in palate	R subtotal maxilla	1	No
3	68	M	Squamous cell carcinoma in upper gingiva	R subtotal maxilla	2	Yes
4	61	M	Squamous cell carcinoma in maxilla sinus	R maxilla	1	Yes
5	58	F	Pleomorphic adenoma in palate	R maxilla	1	No
6	52	M	Squamous cell carcinoma in upper gingiva	L subtotal maxilla	1	No
7	44	M	Squamous cell carcinoma in upper gingiva	L subtotal maxilla	1	No
8	50	F	Osteosarcoma in maxilla	R maxilla	1	No
9	40	M	Osteofibroma	L subtotal maxilla	1	No
10	70	M	Squamous cell carcinoma in maxilla sinus	R maxilla	1	No
